# Successful Treatment of Central Nervous System Histiocytic Sarcoma With Craniectomy and Adjuvant Radiotherapy

**DOI:** 10.7759/cureus.24690

**Published:** 2022-05-03

**Authors:** Soroush Shahrokh, Afshin Rakhsha, Mohadese Shahin, Amir Javadzadegan, Mahsa Ahadi, Samira Azghandi, Farzad Taghizadeh-Hesary

**Affiliations:** 1 Graduate Medical Education, Hospital Corporation of America (HCA) Houston Healthcare Kingwood/University of Houston School of Medicine, Kingwood, USA; 2 Department of Radiation Oncology, Faculty of Medicine, Shohada-e Tajrish Hospital, Shahid Beheshti University of Medical Sciences, Tehran, IRN; 3 Department of Radiation Oncology, Faculty of Medicine, Iran University of Medical Sciences, Tehran, IRN; 4 Department of Radiation Oncology, Shohada-e Tajrish Hospital, Shahid Beheshti University of Medical Sciences, Tehran, IRN; 5 Department of Pathology, Shohada-e Tajrish Hospital, Shahid Beheshti University of Medical Sciences, Tehran, IRN; 6 Department of Radiation Oncology, Shahid Beheshti University of Medical Sciences, Tehran, IRN

**Keywords:** radiotherapy, radiation therapy, histiocytic cell neoplasm, cns sarcoma, cns histiocytic sarcoma, histiocytic sarcoma

## Abstract

Histiocytic sarcoma (HS) is a rare, aggressive non-Langerhans histiocytic cell neoplasm of hematopoietic origin. Histiocytic sarcoma is prone to early systemic metastasis, rendering early diagnosis and treatment critical determinants for patient outcome. Primary HS originating from the central nervous system (CNS) is exceptionally rare and portends a poor prognosis. This grim clinical course is further complicated by the challenging diagnosis and the lack of standard treatment guidelines for the disease. This is due to the exceptionally rare nature of primary CNS histiocytic sarcoma and the limited data available on the successful management of the disease, prompting the therapeutic approach to be guided by retrospective data from case reports or single-institutional studies with a limited number of patients. Here, we report a case of a young Middle Eastern male who was diagnosed with primary CNS histiocytic sarcoma, successfully treated with frontotemporal craniotomy and adjuvant radiation therapy. We also elucidate the role of the CD163 biomarker in diagnosing HS and using surgery and adjuvant radiotherapy (RT) as a successful treatment approach for primary CNS histiocytic sarcoma.

## Introduction

Histiocytic sarcoma (HS) is a rare hematopoietic neoplasm of non-Langerhans histiocytic cells. The etiology of HS remains unknown, but it is composed of neoplastic cells with morphologic and immunophenotypic features of mature tissue histiocytes [1,2]. Despite its terminology, HS is not a true sarcoma but rather a neoplasm derived from the monocyte-macrophage cell lineage [3]. HS may occur sporadically or concurrently with other hematologic malignancies, particularly with acute lymphoblastic leukemia or follicular lymphoma [3]. To date, less than 200 cases of HS have been reported in the literature. Although HS occurs in both genders and across all age groups, data from the United States Surveillance, Epidemiology, and End Results (SEER) database have shown a median diagnosis age of 63 and moderate predominance in men [4]. HS is an aggressive tumor highly susceptible to early systemic dissemination; therefore, early diagnosis and management are crucial determinants of patient outcomes [4]. Primary central nervous system (CNS) involvement is exceptionally rare and portends a poor prognosis [5-8].

Here, we report a rare case of a 35-year-old Iranian male with primary CNS histiocytic sarcoma involving the parietal and sphenoid bones, successfully treated with surgical resection and adjuvant radiotherapy (RT). Furthermore, we will review the differential diagnosis of HS and describe its clinical, pathologic, and immunohistochemical (IHC) features, including the role of the CD163 biomarker for the accurate pathologic diagnosis of HS.

## Case presentation

**Figure 1:**
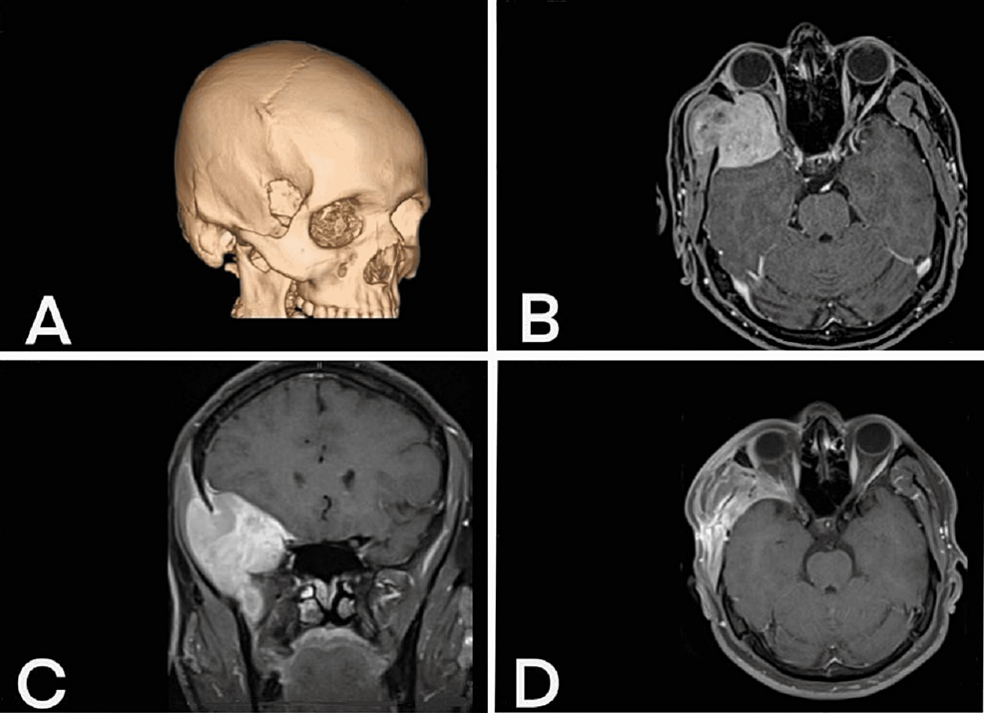
(A) Three-dimensional reconstructed CT scan image showing the destruction of the adjacent sphenoid bone at the superolateral orbit. (B) Axial gadolinium-enhanced T1-weighted preoperative MRI demonstrating lobulated extra-axial heterogeneous enhancing mass at the right frontotemporal region composed of extracranial and intracranial components causing right orbital nerve compression. (C) Coronal gadolinium-enhanced T1-weighted preoperative MRI showing lobulated extra-axial enhancing mass at the right frontotemporal region composed of extracranial and intracranial components. (D) Axial gadolinium-enhanced T1-weighted postoperative MRI showing soft tissue swelling and edema at the superolateral orbit and temporal fossa.

A 35-year-old Iranian man was referred to the outpatient neurosurgical clinic for evaluation of a right cranial mass. The patient initially presented with a chief complaint of chronic, gradually worsening dull headache and swelling of the right parietal bone and orbit, which progressively enlarged in a month into a large lesion measuring approximately 4 × 7 cm. He had no past medical history and denied any familial cancer history. Preoperative computed tomography (CT) scan (Figure 1A) revealed a large right extra-axial frontotemporal mass, causing the superior and lateral orbit destruction. Subsequent magnetic resonance imaging (MRI) (Figure 1B, 1C) revealed a 4 × 7.7 × 4.6 cm (anterior-posterior, sagittal, and transverse, respectively) lobulated extra-axial heterogeneous enhancing mass, with extension into the infratemporal fossa and involvement of the temporal bone and temporalis muscle. The patient underwent frontotemporal craniotomy with total tumor resection. There were no intraoperative complications, and a complete surgical resection with grossly negative surgical margins was achieved. The patient’s postoperative MRI demonstrated right frontotemporal craniotomy with soft tissue edema of the superolateral orbit and temporal fossa consistent with postsurgical changes (Figure 1D).

**Figure 2:**
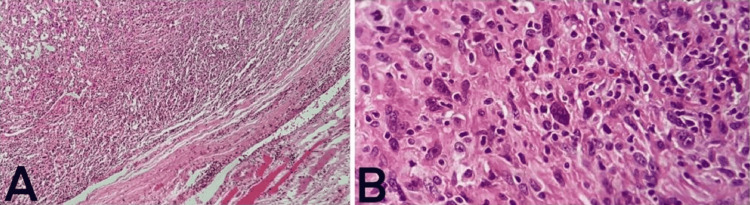
(A) Low magnification of diffuse infiltration of histiocytic cells with a sharp border with the adjacent tissue. (B) High magnification of atypical histiocytes with numerous mitotic figures.

**Figure 3:**
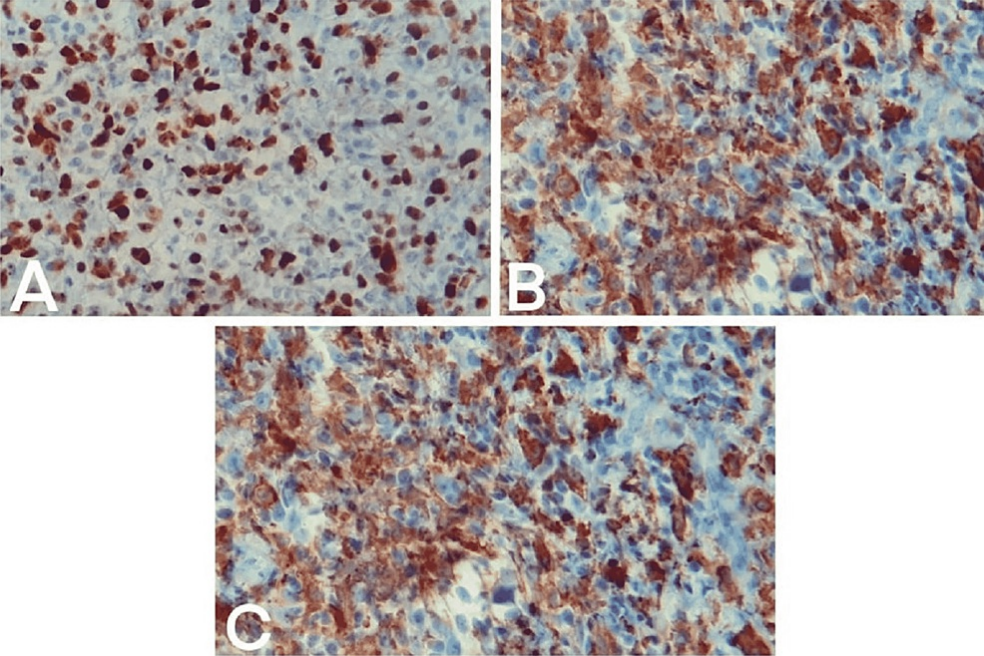
(A) High proliferative index of the tumor cells in Ki67 immunostaining. (B) The tumor cells are CD68-positive on IHC staining. (C) The tumor cells are CD163-positive on IHC.

The resected tissue was sent for pathologic evaluation. Microscopic examination of the specimen revealed diffuse proliferation of the neoplastic histiocytic cells infiltrating normal brain parenchyma (Figure 2A). Hematoxylin and eosin (H&E) staining revealed hypercellular sheets of neoplastic tissue composed of enlarged pleomorphic round to spindle cells with prominent nucleoli, large pleomorphic vesicular nuclei, and eosinophilic cytoplasm; the neoplastic histiocytes showed numerous mitotic figures with no focal necrosis (Figure 2B). Immunohistochemical (IHC) staining revealed tumor cells with diffusely positive expression of Ki67, consistent with a high proliferative index (Figure 3A), cluster of diﬀerentiation (CD) 68, and CD163 (Figure 3B, 3C). Additional IHC panel revealed tumor cells positive for CD3, CD4, CD5, CD7, CD79a, and LCA and negative for S100, HMB45, Melan A, cytokeratin (CK), and LAC, consistent with HS (Table 1). All of the microscopic margins were free of tumor cells. Two expert pathologists separately evaluated the specimen and IHC panel and agreed with the final diagnosis of HS.

**Table 1:** Complete immunohistochemical panel results. ALK1: anaplastic lymphoma kinase-1, CD: cluster of differentiation, CK: cytokeratin, EMA: epithelial membrane antigen, LCA: leukocyte common antigen

Marker	Description of reaction
LCA	Positive in nearly all infiltrative lymphoid cells
CD20	Positive in few scattered small lymphocytes
CD3	Positive in small- to medium-sized lymphoid cells
CD5	Positive in small- to medium-sized lymphoid cells
CD4	Positive in many background histiocytoid cells
CD8	Positive in few scattered small lymphocytes
CD7	Positive in many scattered lymphocytes
CD30	Positive in few scattered transformed lymphocytes
PAX-5	Positive in few scattered lymphoid cells
CD79a	Positive in some cells
S100	Positive in some scattered stellate macrophage, otherwise negative
CD68	Positive in many infiltrative histocytes
CD163	Positive in many infiltrative histocytes
Ki67	Highlights many histocyte nuclei (35% positive)
CD1a	Negative
CD19	Negative
CD15	Negative
ALK1	Negative
CK	Negative
CD23	Negative
Melan A	Negative
HMB45	Negative
EMA	Negative
CKAE1/AE3	Negative
CD21	Negative
CD56	Negative

Postoperatively, the patient was referred to our clinic for adjuvant radiotherapy (RT). Further staging workup with contrast-enhanced CT of the chest, abdomen, and pelvis demonstrated no distant metastasis. His laboratory studies were within normal range, and his bone marrow biopsy revealed no pathologic findings.

**Figure 4:**
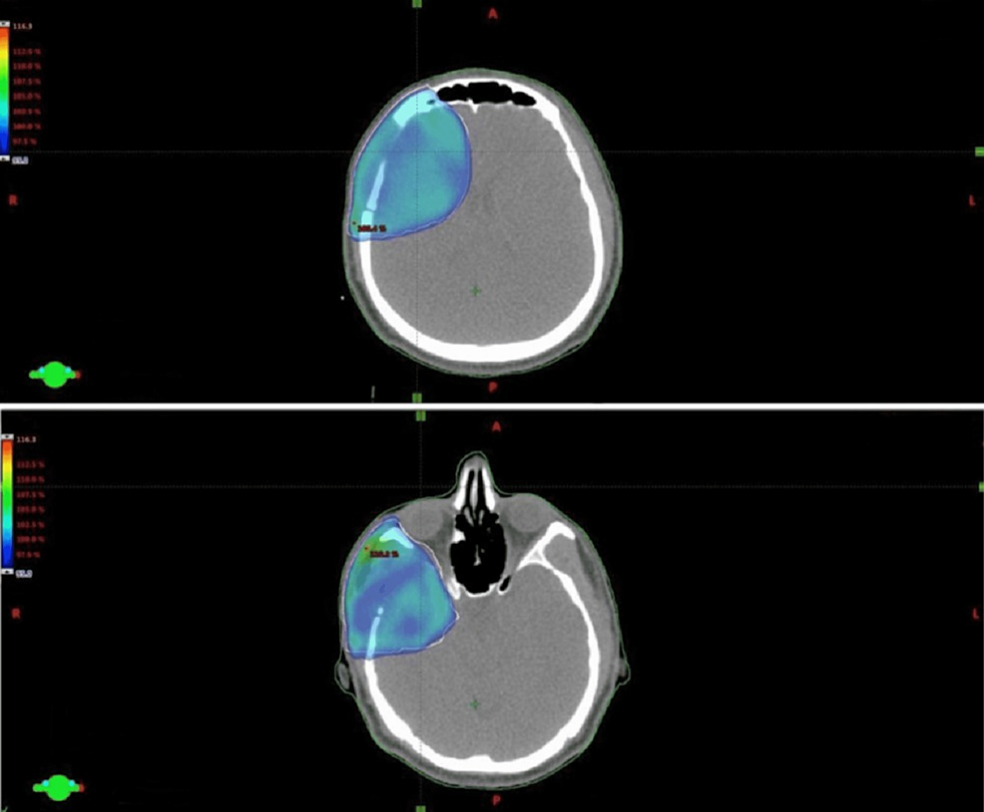
Radiotherapy planning of a case with primary CNS histiocytic sarcoma using the Eclipse Treatment Planning System (TPS).

Because of the high risk of disease recurrence, the patient was treated with prophylactic low-dose adjuvant RT using 50.4 Gy radiation dose in 28 fractions. CT simulation with a radiation mask was used with the patient in the supine position. For contouring and planning, MRI fusion with Eclipse Treatment Planning System (TPS) version 13.5 (Varian Medical Systems, Palo Alto, CA, USA) was used (Figure 4), and for treatment verification, daily images with the electronic portal imaging device (EPID) were obtained. The daily dose of 1.8 Gy was delivered to the patient with Varian Linear Accelerator (LINAC) using the intensity-modulated radiation therapy (IMRT) technique. The patient was evaluated every week for radiation toxicity. His treatment was complicated by mild, self-limited radiation-induced dermatitis (grade 1) and xerophthalmia, successfully managed with topical ointments.

The patient completed adjuvant RT, and his head and neck MRI obtained six months and one year after the completion of adjuvant RT demonstrated no evidence of residual neoplasm or tumor recurrence. The patient remains in complete remission until now.

## Discussion

Histiocytic sarcoma (HS) is a rare, aggressive hematopoietic neoplasm composed of non-Langerhans histiocytic cells derived from the monocyte-macrophage cell lineage [2]. HS predominantly arises in extra-nodal tissues, most commonly in the gastrointestinal, musculoskeletal, and cutaneous organs [9]. The clinical features of HS are nonspecific and predominantly dependent on primary organ involvement. Most patients with HS present with extra-nodal disease symptoms caused by a rapidly enlarging lesion with mass effect [9,10]. Although some patients with primary lymphatic system involvement may present with constitutional symptoms (i.e., fever, fatigue, night sweats, and weight loss), most patients with HS have no systemic symptoms upon initial diagnosis [9,10]. The differential diagnosis of HS is very broad and primarily dependent on the primary organ of origin. The most common differential diagnoses include lymphoma (particularly diffuse large B-cell lymphoma), aggressive meningioma, glioblastoma, malignant Langerhans cell histiocytosis, metastatic melanoma, or dendritic cell sarcoma [11].

Primary CNS histiocytic sarcoma is exceptionally rare, with only 33 cases reported in the literature [5-8]. Definitive diagnosis requires extensive histopathologic and immunohistochemical studies [10-12]. However, owing to its rarity and close histopathologic resemblance with other lymphoproliferative disorders, the accurate diagnosis of HS has proven to be extremely challenging [4,10]. This diagnostic challenge is caused by the broadly similar morphologic features of HS and other lymphoproliferative neoplasms, including B- or T-cell non-Hodgkin’s lymphomas, characterized by large epithelial or pleomorphic cells with prominent nucleoli and vesicular nuclei [10,12].

However, this diagnostic challenge has been largely resolved with the identification of specific HS biomarkers. These include positive immunostaining for CD4, CD45, CD68, CD163, and lysozyme and negative immunostaining for biomarkers expressed by neoplastic T- or B-cells (i.e., CD3, CD5, CD20, CD21, CD15, CD30, CD52, and PAX-5), myeloid cells (i.e., myeloperoxidase and CD33), malignant melanoma (i.e., HMB45 and protein S100), or epithelial cells (i.e., cytokeratin and EMA) [12-14]. Furthermore, recent studies have revealed CD163 as a promising marker for histiocytic neoplasms [14]. These pathologic findings were consistent with our patient’s IHC study results, which revealed positive expression of CD4, CD68, and CD163 biomarkers.

Primary CNS histiocytic sarcoma portends an extremely poor prognosis, with a median overall survival of less than five months after the initial diagnosis [15]. Because of insufficient data, there is no standard treatment approach for primary CNS HS [15]. The treatment approach is often multimodal, including surgery followed by adjuvant chemoradiation [15]. Radical surgical resection, if feasible, is associated with significantly improved prognosis, with an overall disease-free survival period of more than 42 months postoperatively [2,10,15]. Multisystemic disease, or solitary tumor measuring greater than 3.5 cm, on the other hand, is associated with poor prognosis [2,15].

## Conclusions

Histiocytic sarcoma (HS) is a rare, aggressive non-Langerhans histiocytic cell neoplasm with a challenging diagnosis and high susceptibility to early systemic metastasis, rendering early diagnosis and treatment critical elements of patient outcome. Primary central nervous system (CNS) histiocytic sarcoma is exceptionally rare and portends a poor prognosis, further complicated by the challenging diagnosis and the lack of clear treatment guidelines. This has led to a high rate of initial misdiagnosis with delayed initiation of treatment and overall detriment of patient outcomes.

Here, we reported an extremely rare case of a patient with primary CNS histiocytic sarcoma successfully treated with total frontotemporal craniectomy and adjuvant RT. Furthermore, we elucidated the significance of the CD163 biomarker for the diagnosis of HS. HS remains highly chemoresistant, warranting further investigations to determine the optimal treatment modality for the management of HS.
